# Refining animal research: The Animal Study Registry

**DOI:** 10.1371/journal.pbio.3000463

**Published:** 2019-10-15

**Authors:** Bettina Bert, Céline Heinl, Justyna Chmielewska, Franziska Schwarz, Barbara Grune, Andreas Hensel, Matthias Greiner, Gilbert Schönfelder

**Affiliations:** 1 German Federal Institute for Risk Assessment, German Centre for the Protection of Laboratory Animals (Bf3R), Berlin, Germany; 2 German Federal Institute for Risk Assessment, Department Exposure, Berlin, Germany; 3 University of Veterinary Medicine Hannover, Foundation, Hannover, Germany; 4 Charité—Universitätsmedizin Berlin, corporate member of Freie Universität Berlin, Humboldt-Universität zu Berlin, and Berlin Institute of Health, Berlin, Germany

## Abstract

The Animal Study Registry (ASR; www.animalstudyregistry.org) was launched in January 2019 for preregistration of animal studies in order to increase transparency and reproducibility of bioscience research and to promote animal welfare. The registry is free of charge and is designed for exploratory and confirmatory studies within applied science as well as basic and preclinical research. The registration form helps scientists plan their study thoroughly by asking detailed questions concerning study design, methods, and statistics. With registration, the study automatically receives a digital object identifier (DOI) that marks it as intellectual property of the researcher. To accommodate the researchers concerns about theft of ideas, users can restrict the visibility of their registered studies for up to 5 years. The full content of the study becomes publicly accessible at the end of the embargo period. Because the platform is embedded in the infrastructure of the German Federal Government, continuity and data security are provided. By registering a study in the ASR, researchers can show their commitment to transparency and data quality to reviewers and editors, to third-party donors, and to the general public.

## Abstract 2

This Community Page article presents the Animal Study Registry (www.animalstudyregistry.org), which was launched in January 2019 for pre-registration of animal studies in order to increase transparency and reproducibility of bioscience research and to promote animal welfare.

## Introduction

The scientific community is striving for greater transparency in animal research as a measure to enhance the reproducibility of results and to gain more knowledge from animal studies.

Missing efficacy was found to be the main reason for clinical failure of drug candidates [1–4], and irreproducibility of preclinical data was blamed to be the dominating cause. Thus, scientific progress and development of new medical therapies are and will be slowed down by poor quality of preclinical data. The problems regarding the reproducibility of animal studies appear in all bioscientific disciplines studying animals [5]. Therefore, changes are needed to improve the reproducibility within biosciences.

Numerous factors contribute to the irreproducibility of research studies. Biological heterogeneity and complexity as well as the use of nonstandard methods or technologies certainly are the most common reasons for lack of reproducibility [6]. Other key factors impairing the reproducibility of data from biosciences are reporting bias and the low probability to successfully publish “negative” and inconclusive results, hypothesizing after the results are known (HARKing), *p*-hacking, and poor statistical design [7–9]. Misidentification or contamination of reagents, biologicals, and cell lines used have been named as further causes [10].

Dissecting the fundamental structure of a research project can help solve the problems mentioned above. Research projects can be divided into 5 stages: planning, execution, documentation, analysis, and publication. Adjusting each of these steps can significantly refine the whole scientific process. Improving the statistical planning of studies by increasing the statistical power can raise the reproducibility of results by preventing the overestimation of effect sizes and reducing false positive outcomes [11,12]. Lowering standardization in the execution of experiments, for instance, by performing multilaboratory studies, by using different animal strains and sexes, or by diversifying housing conditions, can boost the external validity of research results [13,14]. Transparent documentation and data sharing can help retrace study results and give other researchers the possibility to reproduce experimental outcomes and to build new research questions upon them [15]. The application of a standardized structured quality management system in academic research is a good instrument to identify flaws at all stages of the study [16,17]. The Enhancing the Quality and Transparency of Health Research (EQUATOR) network, for example, provides a comprehensive library of guidelines to assist the health research reporting in various disciplines [18]. The Animal Research: Reporting of In Vivo Experiments (ARRIVE) guidelines are addressing the specific needs for reporting animal research [19]. They were developed by the National Centre for the Replacement, Refinement, & Reduction of Animals in Research (NC3Rs) to maximize the information gained from publications involving animal experiments and thereby minimizing redundant animal experiments [19,20]. Although over 1,000 journals have endorsed the ARRIVE guidelines, their impact has been questioned recently, because the reporting quality has not really improved [21]. As a potential reason for the ARRIVE guidelines’ failure, the IICARus study (a randomized controlled trial of an intervention to improve compliance with the ARRIVE guidelines) identified that requesting the ARRIVE-checklist at the submission stage might be too late within the research process [20].

The Planning Research and Experimental Procedures on Animals: Recommendations for Excellence (PREPARE) guidelines were developed to support scientists already at the stage of planning an animal experiment [22]. It is a checklist addressing different aspects that should be considered before starting an experiment. These include the study design, formulation of a working hypothesis for confirmatory studies, statistical planning, general conditions of animal husbandry and the quality characteristics of test substances, which are also addressed by the Animal Study Registry (ASR). In addition, the PREPARE guidelines focuses on legal and ethical issues as well as on the interplay between the different stakeholders involved in animal experimentation, such as care takers, technical staff, veterinarians, scientists, and facility managers, which are not included in the ASR. In general, the impact of checklists can be discussed, because their use does not necessarily entail a better performance or outcome. Checklists are indeed a good tool to assist clearly defined procedures but might not be sufficient for complex interventions [23]. Supportive measures that require a structured and active involvement of the scientist are more likely to lead to changes in the planning of animal experiments.

We believe that preregistration of research studies can be more effective, because it tackles the problem at its earliest stage and brings researchers to consider all relevant details for a future project in an interactive and self-obliged manner. Preregistration means that researchers describe and register a study design, including statistical planning and method description prior to the first experiment. Being asked distinctive questions, researchers are supported in thoroughly planning their animal experiments. They become aware of important measures to increase data quality, such as randomization and blinding, before they conduct the experiments. In addition, researchers will be stimulated to consider whether the planned study aims to generate or to test a hypothesis. Distinguishing between the exploratory (hypothesis generating) or confirmatory (hypothesis testing) nature of a study can raise awareness on HARKing, i.e., the habit of presenting results based on a post hoc hypothesis as if they were confirming a hypothesis [24]. The transparent description of and adherence to a preregistered statistical analysis plan may also mitigate the effect of tuning test parameters to achieve statistical significance (*p*-hacking). Thereby the credibility in research findings can be regained as the data of a publication become traceable and can be directly related to the original study plan [25]. In the long term, preregistration can help fight the publication bias, i.e., reporting only significant results as they are more likely to get published than negative results [26]. In fact, in clinical research, in which the registration of clinical trials has been mandatory for now more than 10 years, preregistration effectively increased the publication of negative results [27].

There are two ways of preregistration; i.e., study protocols can be registered in a public registry or can be submitted to a journal as a registered report. Advantages of registered reports are the peer-review of the study protocol providing feedback to the author and, with acceptance, the guaranteed publication of the results independently from the outcome [28]. The benefit of preregistering a study plan in an open registry is that the initial protocol can be changed and adapted to new conditions or insights. Thereby scientists are provided with more flexibility regarding the respective research question. An open registry also allows researchers to submit the final results to the most appropriate journal, whereas registered reports bind researchers to a specific publisher.

**Fig 1:**
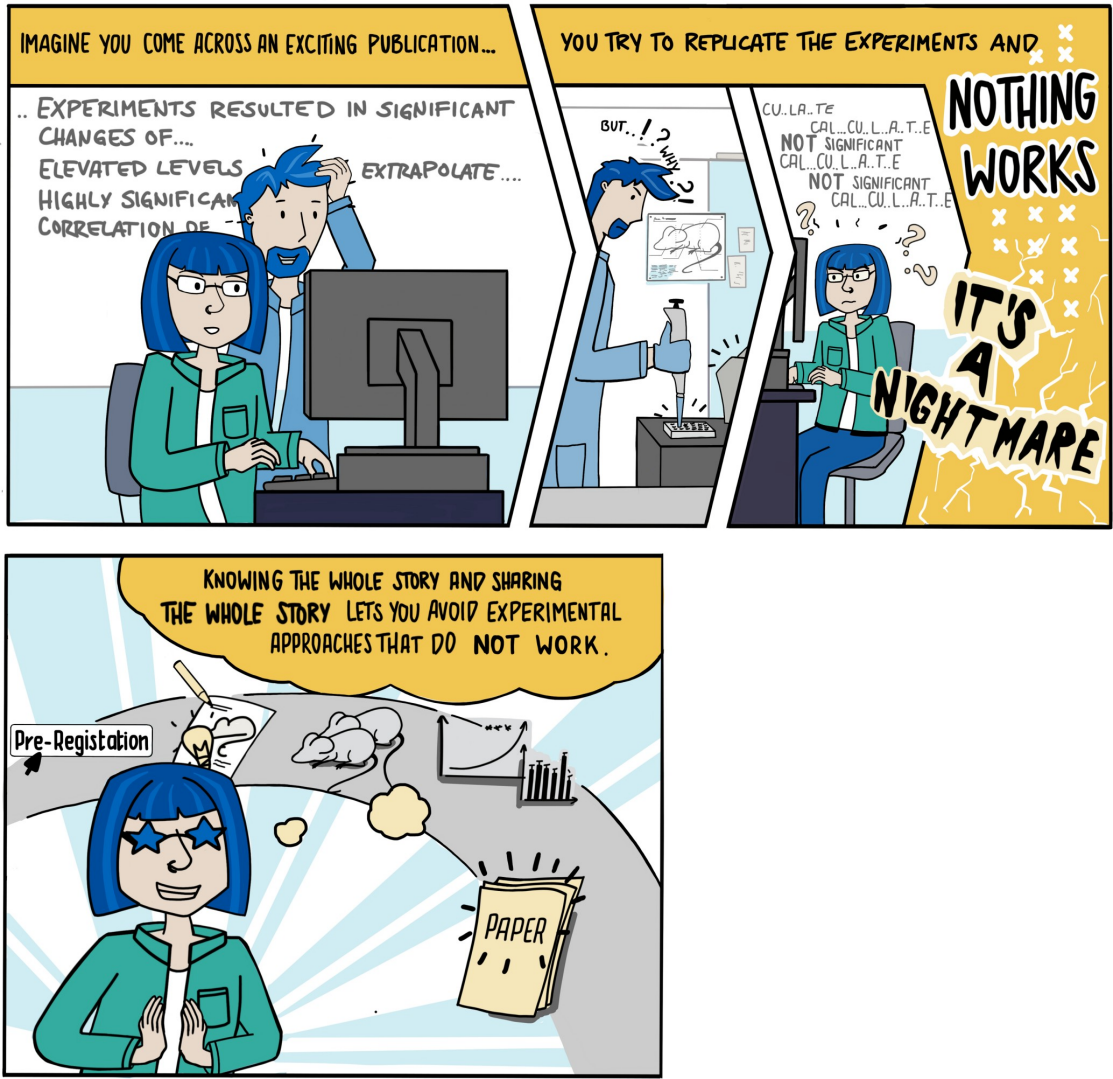
The ASR. ASR, Animal Study Registry.

In January 2019, the German Centre for the Protection of Laboratory Animals (Bf3R) has launched the ASR (www.animalstudyregistry.org; Fig 1), a preregistration platform addressing the particular needs across different research disciplines involving animals. The Bf3R was founded 2015 as a federal institution to coordinate nationwide all activities associated with the goal to provide laboratory animals the best possible protection and to reduce animal experiments to the indispensable minimum. Thus, we highly support initiatives by the scientific community to constantly adjust the quality of research, especially when animals are involved. The intensified discussion about how to implement preclinical registries in animal research [29] brought us to the idea to offer scientists a global platform for preregistering animal experiments. Thereby not only transparency and reproducibility of bioscience research can be improved but also animal welfare can benefit from this measure. The ethical responsibility toward the animals used should lead us to avoid “unnecessary” and redundant animal experiments to our best. The ASR is one of several possibilities to preregister preclinical and basic research projects, such as the registries hosted by the Open Science Framework (osf.io), AsPredicted (aspredicted.org), Research Registry (researchregistry.com), or Preclinicaltrials (preclinicaltrials.eu), though it focuses on animal research. Even though we have experienced a lot of positive feedback since the launch of the registry, we also observe reservations of the scientists against the preregistration of animal studies. Indeed, the number of registrations since January 2019 is still rather low. Up until now, 19 studies by 12 different authors, including one exemplary study provided by the ASR (DOI: 10.17590/asr.0000091), have been registered. It will therefore be a crucial challenge for the future to create incentives for the preregistration of animal studies.

## Registration process of ASR

**Fig 2:**
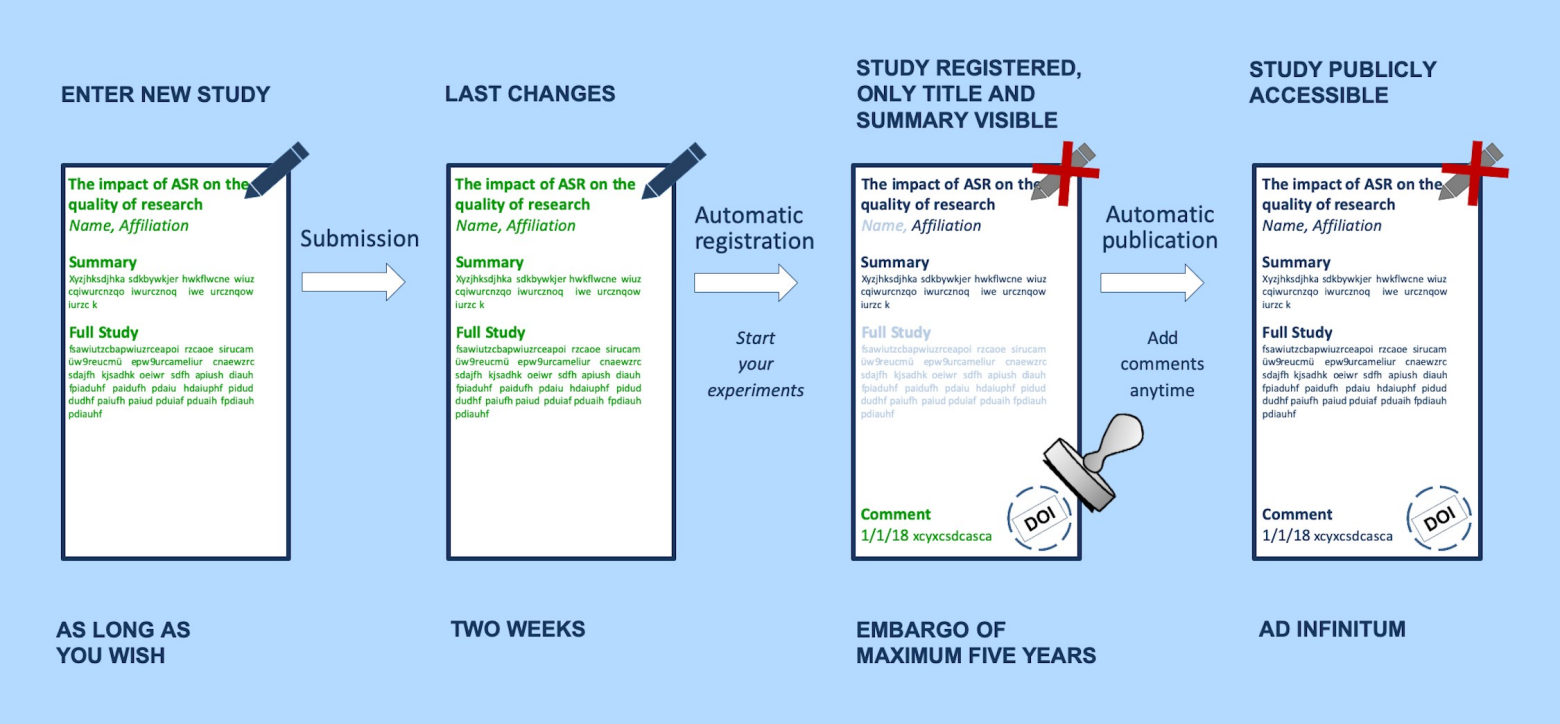
Protection of intellectual property. After submission, the registered study will be under an embargo period of 5 years maximum. Registered studies are only visible with limited content. Once registered, the study automatically receives a DOI. ASR, Animal Study Registry; DOI, digital object identifier.

The ASR registration process consists of four main steps: (1) entering the data, (2) submitting the study, (3) registration of the study, and (4) publication of the study (Fig 2). To register a study, a number of questions about the planned study must be answered. The ASR covers the most important items of the current ARRIVE guidelines, no. 1 through 4 and no. 6 through 13 [19], and it seeks to keep the bureaucratic effort and time costs for registering a study as low as possible. Therefore, the study registration form has a structure similar to other formal documents like grant applications or applications for approval of animal experiments, and is divided into 5 main sections, i.e., general information, study design, methods, statistics, and animal characteristics (see Box 1). The time of data entry into ASR can vary considerably and depends on the experience of the individual scientist and the planning status of the study. An experienced scientist, who has already developed a study plan and is preparing a grant proposal or application for approval of an animal experiment, will need about 2 to 4 hours to enter and double-check the requested information. This seems like a lot of time, but it is well invested when thinking about saving time when writing the paper or wasting time on conducting a less elaborated experiment. After submitting the completed form, the researcher can stop the registration process within 2 weeks by reediting the respective study. If the study remains unchanged, it will automatically be assigned as registered after 2 weeks and concurrently receives a Digital Object Identifier (DOI by DataCite). The ASR does not provide a peer-review process. However, each study will be checked for meeting the basic requirements of the ASR, such as English language, the involvement of animals, absence of offensive contents, and meaningful contents of all filled-in fields.

**Box 1. The structure of the study registration form**

**Preregistration in the ASR**

**General information**

This section gives a short overview of the study:

- Study title
- Short summary of the study
- Author’s name and affiliation

**Study design**

A detailed description of the study design is crucial for preregistration. We encourage researchers to make the description of the study design as transparent as possible, e.g., using timelines, providing a clear definition of the experimental and the control groups, parallel groups or cross-over design, and indicating if animals have been used in previous procedures. The following aspects can be considered essential for a transparent preregistration of a study design:

- Confirmatory studies: A clear hypothesis must be provided.
- Exploratory studies: Because of the nature of the study, it is often not possible to state a clear hypothesis, thus, the research question should be specified as much as possible.
- Free text fields are available to describe legitimate modifications of these classical approaches, e.g., when one study serves both exploratory and confirmatory purposes.
- Method of blinding
- Method of randomization.
- If no blinding and/or randomization will be applied, the reasons should be briefly stated.

**Methods**

Multiple methods can be entered in the preregistration form.

All details of the used methods necessary for the interpretation and replication of the results shall be provided; this may include the following:

- Details of the apparatus
- Description of the consumables including the supplier
- Software used for analysis
- Time of the day when the experiments will be conducted
- Measured parameters and their respective unit
- Previous handling or training of animals
- Method of euthanasia in case of ex vivo studies
- Details of the narcotic and/or analgesic treatment (e.g., prenarcotic treatment, type of anesthesia including the name of drugs and substances)
- Details of the drugs and substances being used including, e.g., name of the supplier, route of administration, dosing, treatment intervals, duration and time point of the treatment
- Provision of the suppliers’ name and catalog number if antibodies will be used
- A list of all cell lines, viruses, DNA or RNA constructs, and bacteria that will be used. For all cell lines, provision of the source of the cell line, authentication, and tests for mycoplasma contamination. If possible, use the respective standard nomenclature.

**Statistics**

Multiple statistical analyses can be indicated in one preregistration entry and connected to the respective experimental method.

At least the following aspects in should be included:

- The main experimental endpoints the sample size calculation relies on
- A sample size calculation
- Any additional outcomes measures that will be assessed
- What kind of primary statistical analysis will be used
- Exclusion criteria for certain data points if applicable

**Animals**

Being able to identify which animal strain is used in an experiment is a crucial aspect of reproducibility in animal research. The exact nomenclature of the strain or breed, including the name of the breeder or supplier, should be specified. For genetically altered animals, international nomenclature standards should be used, and the type of the genetic manipulation should be briefly described.

Please also provide further information about the animals:

- Sex, age or age range (in days/month),
- Body weight (in g or kg) or where applicable, size, length, or height of the animals (in mm or cm) at the beginning of your experiments.

This list can be complemented with any other information concerning animal characteristics relevant for the reproducibility of the study.

As living or housing conditions also may have a big impact on the study outcome, the ASR preregistration form asks you to provide information on the following aspects of animal living or housing conditions:

- Husbandry type (e.g., natural habitat, conventional, barrier, individually ventilated cage [IVC], specific-pathogen-free [SPF])
- Dark/light cycle (e.g., lights on/off/none, light intensity in lux, season)
- Humidity and temperature (in % and°C, respectively)
- Single housing/group housing (e.g., if single housing, specify for how long; number of animals per cage, separated by sex, natural social structure)
- Cage/tank type and size (e.g., width × depth × height)
- Bedding material (e.g., type, name of supplier)
- Environmental enrichment (e.g., nesting material, animal houses, toys, special food, plastic plants)
- Cage changing practices (e.g., cage changes per week, handling of animals)
- Feeding practices (e.g., type of food, name of supplier, irradiated or autoclaved)
- Water quality/supply (e.g., fresh water, tap water, acidified water, for fish: Water changing practices)

Information on any refinement measures implemented in the research project should be provided.

Any additional information you consider important for the reproducibility and transparency of your study can be added. Furthermore, every section offers the possibility to upload additional data.

The intellectual property and the associated theft of ideas are major concerns brought forward by scientists [29]. To address this issue, the ASR provides an up to a 5 year embargo, during which the study is only publicly visible with the title and a short summary, the name of the institution, and, optionally, the name of the researcher. However, a complete PDF version of the registered study can be downloaded by the author at any time. The PDF file containing all study details and metadata can be submitted together with a manuscript or a grant application disclosing the full study plan to the reviewers and proving the study registration, even though the study is still under embargo. After the embargo has expired, the full contents of the study will become visible and searchable for everyone. Anyone can browse the database without being registered. Although the contents of a registered study cannot be changed, the ASR offers the possibility to add comments anytime. This allows researchers to accommodate and to explain modifications to the original study plan that became necessary during the research process.

## Different types of research

Every researcher worldwide can use the ASR to register any kind of study involving any animal species, whether it is a vertebrate or invertebrate or an in vivo or ex vivo experiment. The ASR asks researchers to classify their study as either a confirmatory or an exploratory research project. Confirmatory studies that test a priori hypotheses usually derived from a theory based on previous information are particularly suitable for preregistration [12,30]. In this case, a clearly stated hypothesis is essential [12]. In the case of exploratory research, the research question is rather based on data exploration [31], which entails a different type of study planning. Mostly, only a few series of small experiments are conducted and the results thereof give the direction to the following experiments. Consequently, the registration of a detailed large experimental plan stretching over several years might not be feasible. However, exploratory research forms the main part of basic biomedical research and is strongly affected by publication bias and insufficient reproducibility [11]. Thus, we highly encourage the registration of exploratory studies to ensure that all gained results will be published and no information is lost. It must be kept in mind that exploratory studies can also have a statistical analysis plan. The preregistration of such a statistical analysis plan is important to distinguish postdiction from prediction [25]. Postdiction means that explanations for the results are formulated after the data are known, whereas prediction entails that the statistical strategy is formulated before data collection. The exploration of data after their collection is a valuable part of exploratory research that should aim to generate new hypotheses that can be tested in a new set of experiments. However, post hoc explanations are often overestimated because they are perceived and reported as foreseen. To increase the informative value of the statistical outcome, the analysis plan should be preregistered because this allows a clear cut between post- and prediction [25].

To adequately represent the exploratory research process in the ASR, it might be appropriate to submit multiple preregistrations, each of them covering a part of the research project. The ASR allows the duplication of a registration entry with a new title. Text fields remaining the same for all parts of the study do not have to be filled in again (e.g., author, background of the study, general information, etc.), and researchers can simply modify only the necessary parts. If several studies are linked or form a major project, it should be reflected in the study titles, e.g., as part 1, 2, 3, or as pilot and follow-up study.

Furthermore, in each section, free text fields are available to describe legitimate modifications of these classical approaches, e.g., when one study serves both exploratory and confirmatory purposes. It is expected that a description of an adequate statistical analysis in such cases will be provided.

## Updating a study registration in ASR

Research studies, especially exploratory studies, can be performed over a long period and often the course of the experiments cannot be predicted in detail. Thus, the ASR offers the possibility to add comments to a registered study to state or to explain any kind of changes to the original study plan. The comment feature helps reflect the full experimental process and include information potentially beneficial for other researchers, such as methodological dead-ends.

Because most scientific publications only report successful experiments and do not contain any information about what went wrong in the research process, registries like the ASR become an important tool to save this information for the scientific community. This can prevent the repetition of mistakes as well as unnecessary animal experiments that will not deliver the envisaged outcome.

With the completion of the study, the comment feature can also be used to refer to the final or preprint publication(s), to other registered studies, or to data sets stored in open access repositories.

Each submitted comment will be published with a date together with the contents of the original entry.

## Intellectual property and data security

One major concern brought up when preregistration is mentioned is the possible theft of ideas if full contents of a study become visible long before the publication of the data. To address this issue, the ASR offers the possibility to restrict the visibility of an entry for up to 5 years. During this time, only the title and a short summary of the study as well as the name of the institution and, optionally, the name of the researcher are publicly visible. However, for the entire embargo period, the researcher can download a PDFcopy of the registered study containing all study details and metadata that can be attached to a manuscript submitted for publication. Thereby peer reviewers are able to compare the original research protocol with the submitted manuscript. The timespan of 5 years was chosen on the internal experience concerning the average period of research funding as well as duration of research projects. In addition, every study registered in the ASR automatically receives a DOI by DataCite, which marks the study as the intellectual property of the researcher. The platform is embedded in the infrastructure of the German Federal Government; thus, continued operation of the platform and data security are provided.

## Benefits for animal welfare

Improving the quality of scientific studies might have been the initial idea behind the development of study registries for preclinical research. However, the resulting benefits for animal welfare are in no way inferior. In fact, preregistration responds to an increasing public concern about animal welfare and the limited usefulness of results gained from animal experiments [32]. Poor reproducibility of animal experiments does not only damage the credibility of science but it also raises concerns of unjustified use of experimental animals. With the preregistration of studies, more complete data about planned and conducted animal experiments will be preserved for the use of the scientific community, and redundant experiments can be avoided. Indeed, null results or experiments that reveal methodological deficits are often not published, which might lead to an unnecessary repetition of the same experiment by different research groups.

With a separate section referring to animals asking detailed questions concerning husbandry and animal characteristics, such as strain or breed, genetic manipulations, age, sex or body weight, researchers have to consider all factors influencing animal welfare and the experimental outcome already when planning their experiments. Sharing experience about housing, handling, and refinement measures can accelerate the progress in improving laboratory animal welfare.

## Advantages for scientists

Reliable and transparent data are essential to make the scientific and public discourse on animal experiments more transparent. As an early measure within the scientific process, preregistration of studies can raise researchers’ awareness of reporting bias, HARKing, *p*-hacking, and poor statistical design.

Although the primary goal of the ASR is to improve the quality of bioscience research involving animals, it also promotes animal welfare. Preregistration may increase the chance of publishing “negative” results and thereby reducing the publication bias. Nondisclosure of negative results hampers the progress of the entire biomedical research, though its consequences are particularly serious in research using animals. Here, leaving out information may lead to unnecessary duplication of experiments and to wasting animal lives.

The creation of new incentives could help preregistration to prevail in the future. However, the number of preregistrations already sharply increased in the last years, especially in disciplines like psychology [33]. By preregistering studies, scientists can show their adherence to transparency and good scientific practice. Preregistration proves thorough planning and can help convince reviewers and funders of the high quality of the study. Filling in the preregistration form can save time at the end of a project not only by avoiding common mistakes in study design and statistical analysis but also by ensuring compliance with the ARRIVE guidelines already at the start of the study. Overall, the preregistration of animal research studies shows researchers’ commitment to open science, transparency, and data quality.

## Abbreviations

ARRIVE: Animal Research: Reporting of In Vivo Experiments
ASR: Animal Study Registry; Bf3R, German Centre for the Protection of Laboratory Animals
DOI: digital object identifier
EQUATOR: Enhancing the Quality and Transparency of Health Research
HARKing: hypothesizing after results are known
IICARus: intervention to improve compliance with the ARRIVE guidelines
IVC: Individually Ventilated Cage
NC3Rs: National Centre for the Replacement, Refinement, & Reduction of Animals in Research
PREPARE: Planning Research and Experimental Procedures on Animals: Recommendations for Excellence
SPF: Specific-Pathogen-Free
